# Comparison of Quality of Life and Learning Success of Adolescents Surviving Cancer and Their Classmates

**DOI:** 10.1007/s13187-019-1472-7

**Published:** 2019-02-13

**Authors:** Éva D. Molnár, Dénes Kovács, Katalin Bartyik

**Affiliations:** 1 Department of Social and Affective Education, Institute of Education, University of Szeged, Petőfi S. sgt. 30-34, Szeged, 6722 Hungary; 2 Department of Pediatrics, University of Szeged, Korányi fasor 14-15, Szeged, 6725 Hungary

**Keywords:** Cancer survivors, Health-related quality of life, Academic success, Fear of cancer recurrence

## Abstract

The aim of this study was to compare the quality of life and school success of adolescent survivors and their classmates. A survey was conducted among 21 cancer survived 12–18-year-old children and 95 of their classmates by using questionnaires covering (a) characteristics of the quality of life; (b) characteristics of the learning process; and (c) level of the fear of cancer recurrence. Significant difference was found in the field of physical and emotional functions but contrary to expected, the members of the control group reported lower values than survivor children. Those children that were teased because of cancer made friends hardly and got involved in social programs with more difficulty. With reference to the level of development of school motivation and the use of learning strategies, it was experienced a significant difference between the two groups only in the field of planning. Our results show that the better the survived children’s general quality of life is the better results they achieve at school. Their learning achievement is influenced to a much bigger extent by social functions than their physical disadvantages.

## Introduction

Over the last decades, the number of cancer survivors among children has increased to a large degree (70–85%) [1]. Treatment is efficient; however, medical conditions affect the child’s physical, psychosocial, and academic functioning [2]. Returning to school may bring hope to the whole family that the child could live a regular life again. Successful school reintegration is based on complex long-term psychological and behavioral investigations and the vital physical, psychological, and social support of the patients [3].

### Cancer and the Pertaining Effects

Cancer exerts an influence on the child, their family, and their broader environment (school) [3, 4], and may lead to cognitive, physical, emotional, behavioral, and social difficulties [5, 6]. While survival after treatment of childhood cancer has increased, survivors can experience late effects as well, which persist at least 5 years after the diagnosis and treatment [7].

Academic failures [8], dropping out of secondary school, and/or higher education is frequent consequences of the listed problems [9]. These might have secondary effects on further life qualities, such as finding a good job in the labor market [10], and they may increase the level of anxiety and uncertainty [7]. Uncertainty is often linked to worry, which is usually experienced as a state of fear, concern, or uneasiness about child’s future experiences and the recurrence of cancer [11].

Despite the negative outcomes described above, the completion of cancer treatment might also have a positive impact. Many resilience factors have been identified to predict positive adjustment: age of diagnosis, family support and family functioning, individual coping style, and environmental variables [12]. Studies highlight enhanced maturity, psychological development, and efficient coping strategies [13, 14], which may help in the adjustment to school and later work, and general problem-solving skills [7].

### School Returning Difficulties

According to previous findings, it is important for children with cancer to return to school as soon as their health condition allows them to [6]. Active presence at school conveys a symbolic message that the child is better, provides hope that there is life after cancer, and helps the child recover self-control over their life [4, 12].

The long-term hospital treatment isolates children with cancer from their home and school environments, and this isolation has an impact on the emotional well-being of the child [5]. Returning to school may increase shyness in children due to changes in their physical appearance and fear of being teased by their schoolmates [12].

In the quality of life of survivors, a healthy physical, social, emotional, and psychological functioning seems to be poorer for childhood cancer survivors compared with peers [3]. Reduced quality of life may affect poor school attendance and low academic achievement, since due to extended absences students may not be able to keep up their peers academically, and the side effects of the illness (i.e., fatigue) may keep the child from participating in academic and social activities [3, 12].

Much of the quantitative and qualitative research focuses on survivors’ health-related quality of life and their academic and psychosocial functioning [15], but little is known about survivors’ use of learning strategies and learning motives and how these correlate to the long-term effects of cancer. Several studies of the healthy population revealed that effective learning strategies (e.g., metacognitive strategies—planning, monitoring, regulation) and learning motivation (e.g., self-efficacy beliefs, performance approach, mastery motivation) are crucial for successful learning and academic performance [16].

### The Role of the School Environment in Supporting Readjustment

Teachers and parents exert an influence on the successful school reintegration of the survivor. The role of the teacher is focused on creating an inclusive atmosphere in class and an open medium, where the handling of the situation can be achieved more naturally (restraint, over-precaution, and avoidance of extreme tact which hinder both the acceptance of the child and the inspiration of the child’s motivation) [17]. Research done with teachers indicates that the majority of the educators are open and they even need information on the handling of the illness and the consequences by cooperating willingly with the physician and the psychologist [18]. The role of the parents is of paramount significance since their support is determining in this situation. If the parent is overprotective of their child, it makes the return to school more difficult and it might even elicit school phobia from the child [17].

## Purpose of Study

This study has three aims: (1) to examine the level of quality of life of survivors from self-report perspective, and to compare with their classmates’; (2) to test the effects of the individual features (type of cancer, finished treatment time—1, 2, and 5 years, fear of cancer recurrence, teasing experiences); (3) to examine the differences in their academic success, learning strategies, and learning motivations. It was hypothesized that the HRQL scores of survivors would be lower than those of healthy classmates; the type of cancer would have an impact on HRQL (children who have survived a brain tumor would have lower academic and school function); the fear of cancer recurrence and teasing experience would have negative effect of the level of HRQL. Furthermore, there is little information about the finished treatment time—how different are effects on survivors’ HRQL and we have even less information about the features of learning motives and learning strategies among survivors. Our study is explorative in these aspects.

## Methods

### Study Design

This is a case-control study which was carried out in the period between January and March 2013. It includes all adolescents from the ages of 12 to 18 years who were diagnosed with cancer and treated from January 2007 to December 2010 in a small city in East Hungary, and who survived at least 1 (*N* = 8), 3 (*N* = 7), or 5 (*N* = 6) years after finishing treatment. Data were collected by using questionnaires sent to the child’s families. The control group was selected from the classes of the survivors randomly (from the list of survived children in alphabetical order, every fifth child’s class was involved in the investigation). Questionnaires were sent to these students with after the permission of their parents.

### Participants

In the case group, 21 adolescent survivors have participated (girls = 43%; boys = 57%; age mean = 16.22). The biggest proportion of survivors who took part in the study had different types of leukemia and lymphoma (33–33% respectively); next are children recovering from brain cancer (14%), bone cancer (10%), soft tissue sarcoma (5%), and neuroblastoma (5%).

In the control group (*N* = 95), selected from the classes of the survivors were females 69 (73%) and males 26 (27%). The mean age group of the students is 16.53 years; there is no significant difference between the age of survivors and controls.

### Instruments

#### Pediatric Quality of Life Inventory™ – PedsQL™

The Hungarian version of Pediatric Quality of Life Inventory™ – PedsQL™ [19] for the 13–18 age group [20] was used to measure the health-related quality of life (HRQL). There are four domains: physical, emotional, social, and school, altogether 23 items. The PedsQL™ measures HRQL in patients perceived in the previous month. Child self-report tools were used (survivors: Cronbach’s *α* = 0.82; control group: Cronbach’s *α* = 0.88). A Likert scale ranging from 0 to 4 was adjusted (0 = never, 4 = almost always). The items were reverse scored and linearly transformed to a 0 to 100 scale (0 = 100, 1 = 75, 2 = 50, 3 = 25, 4 = 0), where higher scores indicated higher HRQL [19].

#### Fear of Cancer Recurrence Questionnaire (Progredienzangst-fragebogen)

The Fear of Cancer Recurrence Questionnaire (FCRQ) is a 12-item scale assessing worries related to cancer recurrence validated by Herschbach [21]. In Hungarian version, one item related to adults was discarded (11 items remained, Cronbach’s *α* = 0.91). The questionnaire items consist of fears related to the cancer (i.e., intrusive thoughts associated with cancer recurrence, worry related to repeated medical consultation, emotional disturbance associated with cancer recurrence) on a 5-point Likert scale (1 = never, 5 = always). The higher scores indicated higher level of fears. The FCRQ was used only in the case group.

#### Learning Motivation and Learning Strategies

The Self-Regulated Learning Questionnaire (SRLQ) [16] was used to measure learning motivation and learning strategies. The SRLQ is an 88-item self-report questionnaire rated on a 5-point Likert scale (1 = not typical, 5 = entirely typical). There are two subscales: learning motivation subscale (46 items, Cronbach’s *α* = 0.82; six different motives: mastery motivation, performance approaching, performance avoiding motivation, anxiety, positive and negative self-efficacy) and learning strategy subscale (42 items, Cronbach’s *α* = 0.90; eight strategies: memorization, elaboration/organization, planning, monitoring, time management, effort regulation, help-seeking, and procrastination). The SRLQ has a high internal consistency and a good reliability and has been validated for healthy children and adolescents from 10 to 18 years [16]. The higher scores in learning subscales mean more frequent use of learning strategies, and higher scores regarding learning motives indicate higher levels of learning motivation.

#### Background Items

There were two background items regarding school success (grade point average (GPA) ranged from 1.0 = failed to 5.0 = very good, collected GPA for survivors pre- and post-cancer; how satisfied is she/he with school performance—1 = very unsatisfied, 5 = very satisfied) and one item related to teasing experience (was she/he teased by others because of cancer—yes/no, only in survivors).

## Results

### Characteristics of Health-Related Quality of Life

The mean HRQL total score was 75.8 ± 11.8 in survivors and 72.3 ± 12.5 in the control group; there was not significant difference (Table 1). The lowest score was school functioning in survivors, emotional and school functioning in the control group. Significant differences were found in two subscales: physical functioning (survivors 83.78 ± 12.83 vs controls 77.11 ± 14.98; *t* = 2.081, *p* = 0.05) and emotional functioning (survivors 71.67 ± 19.45 vs controls 60.92 ± 18.10; *t* = 2.313, *p* = 0.03). Survivors report significantly higher physical and emotional functions than controls. No significant differences were found in social and school functioning.

**Table 1:** Health-related quality of life in survivor and control group

PedsQL™ scale	Survivors *N* = 21 Mean (SD)	Controls *N* = 92 Mean (SD)	*T* test	
			*t* values	*p* values
Physical function	83.8 (12.8)	77.1 (15.0)	2.081	0.05
Emotional function	71.7 (19.5)	60.9 (18.1)	2.313	0.03
Social function	81.0 (16.9)	85.0 (15.0)	−0.978	0.34
School function	68.8 (17.6)	66.0 (15.9)	0.664	0.51
Total score	75.8 (11.8)	72.3 (12.5)	1.188	0.24

#### Type of Cancer and HRQL

The highest mean HRQL total score was found among children treated with soft tissue, neuroblastoma, and lymphoma (Table 2). The lowest total score was found in the case of children treated with brain tumor. Although it is limited to compare all groups and post hoc tests are not performed because there are two groups fewer than two cases, it can show the differences. The lowest scores were found in emotional, social, and school functioning among children treated with brain tumor, neuroblastoma, and leukemia.

**Table 2:** Cancer diagnoses and HRQL

Type of cancer	N	Physical function	Emotional function	Social function	School function	Total score
		Mean (SD)	Mean (SD)	Mean (SD)	Mean (SD)	Mean (SD)
Leukemia	7	84.8 (12.3)	58.6 (22.3)	83.6 (16.0)	68.6 (20.1)	73.9 (14.2)
Lymphomas	7	85.7 (11.9)	80.7 (13.0)	87.5 (10.8)	73.6 (13.8)	80.7 (10.3)
Bone	2	70.3 (6.6)	77.5 (17.5)	77.5 (24.7)	65.0 (7.1)	72.6 (10.7)
Brain	3	77.1 (17.2)	78.3 (20.2)	56.7 (12.6)	50.0 (20.0)	65.5 (4.7)
Soft tissue sarcoma	1	100.0 (−)	85.0 (−)	90.0 (−)	85.0 (−)	90.0 (−)
Neuroblastoma	1	93.8 (−)	55.0 (−)	95.0 (−)	85.0 (−)	82.2 (−)

#### HRQL in Adolescents Finished Treatment in Different Times (1, 3, and 5 Years)

Based on the ANOVA and Duncan^a,b^ tests, significant differences were found in social function ({1},{3} > {5},{3}, *F* = 3.907, *p* = 0.04), in physical function ({1},{3} > {5},{3}, *F* = 2.386, *p* = 0.03), and in school function ({1},{3} > {5}, *F* = 3.308, *p* = 0.05). Survivors who finished treatment 1 year before have a significantly higher social function (90.0 ± 10.8) and higher physical function (88.0 ± 12.4) than those who finished treatment 5 years before (social 66.0 ±  9.6; physical 73.8 ± 6.5) (there is no significant difference between those who finished treatment 3 and 5 years before). Survivors who have finished treatment 1 and 3 years ago have higher school function (1 year 72.5 ± 13.1; 3 years 75.0 ± 19.1) than those whose treatment ended 5 years ago (53.0 ± 14.0).

#### Fear of Cancer Recurrence and HRQL

Strong significant correlations were found between fear of cancer recurrence and emotional function subscale (*r* = − 0.73, *p* = 0.01). The higher survivors’ fear of recurrence is, the lower the emotional function is. The correlations were not significant for HRQL total score and the other subscales.

#### HRQL in Survivors With and Without Teasing Experiences

Because of the small sample size, Mann-Whitney probe was used to explain the HRQL scales’ differences between survivors with and without teasing experiences. We found significant differences only in social function (teased *N* = 5, 66.00 ± 18.84; non-teased *N* = 16, 86.00 ± 13.39; *Z* = 0.028, *p* = 0.03). Those who were teased because of cancer have a lower score in social function than those who were not teased.

### Academic Performance, Learning Strategies, and Learning Motivation

Significant differences were found between cancer survivors’ pre- and post-cancer academic performance (*t* = 19.57, df = 19.00, *p* = 0.00). Survivors’ GPA is lower post-cancer than before (Table 3), and at the same time, survivors’ post-cancer GPA is significantly lower than in the healthy group (*t* = 2.22, df = 16.00, *p* = 0.04).

**Table 3:** Survivors’ and controls’ academic performance (GPA)

Sample	*N*	Mean	SD
Survivors, pre-cancer	20	4.05	0.93
Survivors, post-cancer	18	3.84	0.85
Control group	87	4.19	0.66

Comparing the satisfaction with academic achievement, no significant difference can be found. At the same time, both the median (survivors 4, controls 3) and the skewness index (survivors − 0.51, controls − 0.10) show that survivors are more satisfied with their academic performance than controls, even if their GPA is significantly lower.

Among the seven categories of learning strategies, only the planning showed significant difference (survivors 38.9 ± 18.5, controls 52.5 ± 21.3, *t* = − 2.96, *p* = 0.01). Survivors use planning strategy less than controls. No significant differences were found in motivation components.

#### Predictions of Academic Success (GPA)

To address predictions of academic success in survivors, we performed a linear regression analysis of GPA on HRQL, learning strategies, and learning motivation (Table 4). We used only those items which showed stronger correlations with GPA, thus from HRQL—the school function (*r* = 0.57, *p* < 0.05), from learning strategies—the time management (*r* = 0.54, *p* < 0.05), from motivations scale—the performance approach motivation (*r* = 0.64, *p* ≤ 0.01). We used the Enter method, when the collinearity indices were all within the proposed thresholds (correlation coefficients below 0.60, VIF 1.1–1.6, tolerance 0.63–0.97). In the first step, we found that survivors with a higher school function received better grades, which explained about 57% of the variance in GPA (see model 1). Moreover, we found that a better time management learning strategy was associated with better GPA (see model 2). However, after including the motivation component (performance approach) into the regression model, the effect of school function and time management turned non-significant (see model 3). Altogether, the three predictors explained 76% of the variance in survivors’ GPA. Regarding the controls, the regression model shows lower values; however, the models are significant, prediction variables explained only 17% of the variance in GPA.

**Table 4:** Regression models of GPA on HRQL, learning strategies, learning and motivation

Dependent variable: GPA	Survivors					Controls				
Predictors	*β*	*p*	*R*^2^	*F*	*p*	*β*	*p*	*R*^2^	*F*	*p*
Model 1			0.57	7.59	0.01			0.08	6.32	0.01
School function	0.57	0.01				0.25	0.01			
Model 2			0.71	7.46	0.01			0.17	7.64	0.00
School function	0.47	0.03				0.22	0.04			
Time management	0.43	0.04				0.31	0.01			
Model 3			0.76	6.32	0.01			0.17	5.21	0.00
School function	0.30	0.18				0.22	0.04			
Time management	0.34	0.09				0.33	0.00			
Performance approach	0.35	0.14				− 0.08	0.50			

Survivors *N* = 21, controls *N* = 95

## Discussion

In our study, adolescents surviving cancer reported higher physical and emotional functions compared with healthy controls. The type of cancer, the fear of cancer recurrence, and teasing experiences have related to different dimensions of HRQL. Our findings revealed that cancer survivors experienced disturbances during cancer, and prolonged absences from school affected their academic performance.

### Survivors’ and Their Classmates’ Health-Related Quality of Life

Even though researchers found unequivocal results in HRQL [22], our findings revealed other aspect of cancer affectation on children’s physical, emotional, and social functions. Significant differences were found in two HRQL functions: physical and emotional functions, but contrary to expected, the survivors showed higher level than the controls. Our findings are inconsistent with other researches [23], which reported low HRQL scores in survivors compared with healthy children. The novelty we introduced is that the healthy sample is chosen among the survivors’ classmates (i.e., the controls and the survivors attend the same class in school), while in the previous studies, there was no relationship between the two groups. Our findings suggest the possibility that participants of the two different groups in our study represented a different conception of illness and “problems.” This is consistent with Macartney et al.’s findings [13] that unpredictability and uncertainty experienced by cancer survivors inspire them to value new opportunities and to learn to be thankful and appreciative of what they already have.

### Survivors’ Individual Differences in Health-Related Quality of Life

A series of studies reported that type of cancer, treatment time, fear of cancer recurrence, and teasing experiences affected different levels of HRQL [13, 22–24].

Our findings are consistent with previous studies, which reported different levels of HRQL for different types of cancer [7]. Our results indicated that adolescents who survived brain tumor had the lowest social and school functioning. These findings show that the type of cancer and the recommended treatments lead to feelings of distress and psychosocial and academic limitations.

New in our study is analyzing HRQL levels from post-treatment period. Our findings showed significant differences in social, physical, and school functions based on finished treatment time. Survivors who have finished treatment 1 year before have a significantly higher social function and higher physical function than those who finished treatment 5 years before, and those who finished treatment 1 and 3 years before have higher school function compared with those whose treatment ended 5 years before. These results suggest that a more recent treatment experience generates more appreciative positive social and physical signs and academic success in everyday activities.

Fear of recurrence is one of the most common psychological phenomena among survivors, and may lead to a set of negative behavior changes (e.g., making plans for the future) and psychological problems (e.g., depression, distress) [24]. Our findings are consistent with these results; we found a strong correlation between fear of cancer recurrence and emotional function. The higher level of fear of recurrence is related to the lower emotional function, which means survivors in our study who worried about the cancer returning have different emotional problems (fear, feelings of anger and aggression, negative mood).

Consistent with findings by others [11], we found significant correlations between teasing experience and HRQL. Survivors teased by others of cancer had significant lower social function than non-teased, i.e., teased survivors in our sample had difficulties adapting to social events and creating relationships. These findings underline the importance of a supportive environment in survivors’ school reentry and social readaptation. Teased survivors in our sample have difficulties adapting to social events and creating relationships over time.

#### Survivors’ and Controls’ Academic Performance

It is important to understand cancer survivors’ academic performance because diminished academic performances can also affect their employment attainment and school outcomes [20]. Consistent with other findings [25], survivors in our study showed diminished academic outcomes post-cancer compared with their healthy peers.

Our findings revealed that variables such as school function and time management mediate the positive impact of academic performance. Our research results are consistent with previous studies [25] which suggest that most cancer survivors are motivated to succeed academically, performance approach motivation showed the strongest correlation with GPA. Regarding the learning strategies, planning was worst in survivors than in their classmates, which is consistent with other findings [24]. It seems that the survivors in our study are uncertain about making plans for the future and this also affected their learning activities.

### Study Limitations

Limitations of this study include the sample size, as only a few survivors were represented in different groups of cancer, which may limit the way our findings can be generalized. Other limitation of our study was that the research was conducted at one particular region, and as such may not be representative of other regions. Furthermore, there were many differences in adolescence during the 12–18-year period. As they were treated together, the conclusions that can be drawn are too broad. As it is worthwhile in the future to assess teasing in more details, other measures for teasing experience (i.e., using scales to measure levels and grades of teasing experiences) should be included, as the binary measure showed to be insufficient. Finally, the quantitative method in itself did not prove to be sufficient, qualitative methods should have been used as well. By using structured interviews, more detailed information could have been found. While we have reported that our research was conducted with a non-representative and small sample, the tendencies we found can possibly provide a clear basis for improving complex reintegration programs in practice.

## Conclusion and Implications

The present study verified differences of health-related quality of life between adolescent cancer survivors and their classmates, and we found higher physical and emotional functions in survivors compared with the healthy group. Our findings suggested that survivors’ physical and emotional functions and learning motivations grow positively because of their negative past experiences. This study showed that school function, learning strategies, and learning motivation are crucial for survivors’ successful learning and good grades. Furthermore, our findings drew attention to the importance of an accepting and supporting school environment which should help survivors’ school reintegration. Teasing and social exclusion may lead to lower social function and problems in social activities. These findings revealed the importance of training programs for teachers working with young cancer survivors, which would facilitate students’ school reintegration and contribute to their academic success and psychosocial development.
